# The role of modern parameters and their relationship with recurrence risk as assessed by Oncotype DX: real-world evidence

**DOI:** 10.3332/ecancer.2024.1664

**Published:** 2024-02-06

**Authors:** Dana Narvaez, Jorge Nadal, Adrian Nervo, Victoria Costanzo, Claudio Paletta, Fernando Petracci, Sergio Rivero, Alexis Ostinelli, Federico Coló, Loza Martín, Veronica Fabiano, Luciana Sabatini, Azul Perazzolo, Mora Amat, Matias Chacon, Federico Waisberg

**Affiliations:** Alexander Fleming Institute, Buenos Aires 1425, Argentina

**Keywords:** breast cancer, recurrence risk, genomic platforms, second primary tumour

## Abstract

Genomic analysis through various platforms is an essential tool for determining prognosis and treatment in a significant subgroup of early-stage breast cancer patients with hormone receptor-positive and human epidermal growth factor receptor 2 (HER2)-negative status. Additionally, combined clinical and pathological characteristics can accurately predict the recurrence score (RS), as demonstrated by the University of Tennessee risk nomogram. In this study, we aimed to identify classical clinical-pathological factors associated with high RS in a local population, including modern parameters such as current abemaciclib treatment recommendations, HER2-low status, different Ki-67 cutoff values, and samples obtained from secondary primary tumours. This is a retrospective single-institution study that analysed a total of 215 tumour samples. Among lymph node-negative patients (*n* = 179), age, Ki67 values, and progesterone receptor status predicted RS after multivariate analysis. HER2-low status was not associated with RS differences (*p* = 0.41). Among lymph node-positive patients (*n* = 36), MonarchE inclusion criteria (15) were not associated with a higher RS (*p* = 0.61), and HER2-low did not reach statistical significance. However, tumours classified as secondary primaries numerically exhibited a higher RS. Based on these findings from our real-world sample, the mere application of clinical and pathological parameters is insufficient to predict RS outcomes. Modern parameters such as HER2-low status or adjuvant abemaciclib recommendations were not associated with RS differences. Regarding the observation of secondary tumours, more evidence is needed to understand whether prior hormone therapy exposure impacts the biological risk of secondary primary tumours.

## Introduction

Approximately 70% of breast cancer patients have tumours that are positive for hormone receptors positive (HR+) and negative for human epidermal growth factor receptor 2 (HER2-). The standard treatment for these patients varies based on the risk of recurrence and includes adjuvant or neoadjuvant chemotherapy and adjuvant endocrine therapy [1]. Within the group of early-stage breast cancer patients with HR+, there is a subgroup with a higher risk of recurrence, determined by clinical and/or genomic criteria [2]. Up to 30% of these patients may experience recurrence within a 5-year period, making them candidates for adjuvant chemotherapy and/or cyclin-dependent kinase inhibitors based on genomic platform results and clinical assessment [3, 4].

The Oncotype DX^®^ somatic study is a prognostic genomic analysis incorporated into American Society of Clinical Oncology (ASCO), National Comprehensive Cancer Network (NCCN), and St. Gallen guidelines, used to guide adjuvant chemotherapy decisions in early-stage breast cancer, providing a recurrence score (RS) result [5, 6]. This platform consists of 21 genes (16 related to breast cancer and 5 reference genes) [7]. In the TAILORx study, the authors demonstrated that patients with a very low risk of recurrence did not derive additional benefits from chemotherapy. For high-risk patients, chemotherapy was found to provide additional invasive disease-free survival benefits. Finally, for patients in the intermediate-risk group (RS 11–25), it was suggested to consider other clinical factors, such as menopausal status and clinical risk, when recommending adjuvant chemotherapy along with endocrine therapy [8]. Subsequently, the RXponder trial, conducted by Kalinsky *et al* [9] reported on the results of 5,083 patients with axillary involvement of one to three lymph nodes. This trial showed that in postmenopausal patients, regardless of lymph node status, chemotherapy could be safely avoided if the RS is less than 26. However, in premenopausal women, chemotherapy demonstrated an absolute benefit of 2.9% in disease-free survival, irrespective of having a low RS.

However, in daily practice, due to access barriers, it is often not possible to use these genomic analysis tools. Therefore, risk calculators based on pathological anatomy elements and clinical patient characteristics also exist. The Tennessee Nomogram, validated using data from 84,339 patients, correlated five clinicopathological variables (age, tumour size, grade, progesterone receptor (PR) status and histological type) to predict genomic risk in Oncotype DX [10, 11]. Histological grade and PR status were the most significant predictors for RS in this test, followed by histological type, tumour size and age [12]. There is an external validation study of the Tennessee Nomogram conducted in Spain; however, there isn’t validation of this tool in the Latin American population. Additionally, in these countries, access to genomic platforms (like Oncotype DX) is more challenging due to economic resource constraints [13].

In this situation, having rigorous clinical information and thereby identifying patients with low genomic risk who could safely avoid Oncotype DX is crucial. The objective of this study was to evaluate whether classical clinical and pathological criteria can predict the risk of distant recurrence as defined by the RS. Additionally, we aimed to assess whether modern parameters such as established risk factors as MonarchE study inclusion criteria (four or more positive nodes, or one to three nodes and either tumour size ≥5 cm, histologic grade 3, or central Ki-67 ≥20%), HER2-low status, and samples from secondary primaries can predict the RS [14, 15].

## Methods

This is a single-institution retrospective study. Data were collected from early-stage HR+ breast cancer patients who underwent tumour genomic testing with Oncotype DX from March 2017 to November 2022. Early-stage breast cancer patients were included according to the definition of localised disease from the St. Gallen consensus, which indicated genomic testing due to the unclear recommendation for adjuvant chemotherapy [26]. Premenopausal patients without lymph node involvement and postmenopausal patients with and without lymph node involvement were included. In our private practice, these patients accessed genomic testing covered by their prepaid health insurance.

The free University of Tennessee calculator was utilised to obtain low and high-risk estimates. The following variables were evaluated using univariate tests, including *t*-tests and chi-square tests, to identify potential predictors in a multivariate model. HER2-low status, breast tumours that were secondary primary tumours, abemaciclib indications according to MonarchE trial criteria, different Ki67 cutoff points (with a maximum of 20%), histological grade, and various values of estrogen and PRs were assessed. Any variable with a *p*-value <0.1 in the univariate analysis was considered a candidate. Multivariate analyses were conducted using logistic regression methods, considering a binary outcome of treatment recommendation in accordance with current treatment guidelines based on RS results. Regarding anatomical and clinical criteria, the criteria taken from Sparano *et al* [2] were used to define clinical high risk. Women under 50 years of age were considered premenopausal since complete menopausal status information was not available in all medical records. We defined Ki-67% cutoff points to assess whether the RS varied according to this characteristic. The evaluation was conducted through a post hoc stratification. The Hosmer-Lemeshow test was used to assess model calibration, and receiver operating characteristic (ROC) curves were constructed to evaluate the discriminative capacity of the regression model achieved. A *p*-value <0.05 was considered statistically significant.

This study was conducted in accordance with the ethical principles and regulations established in the Helsinki Declaration. Informed consent was obtained from the patients, and data protection regulations were respected.

## Results

A total of 215 patients were analysed. The mean age was 53 years (IQR: 46–63), all were female, 16.8% had positive lymph nodes, and 55.3% were in the postmenopausal stage. In Table 1, the clinicopathological characteristics of the patients included in the study are observed.

**Figure 1.**
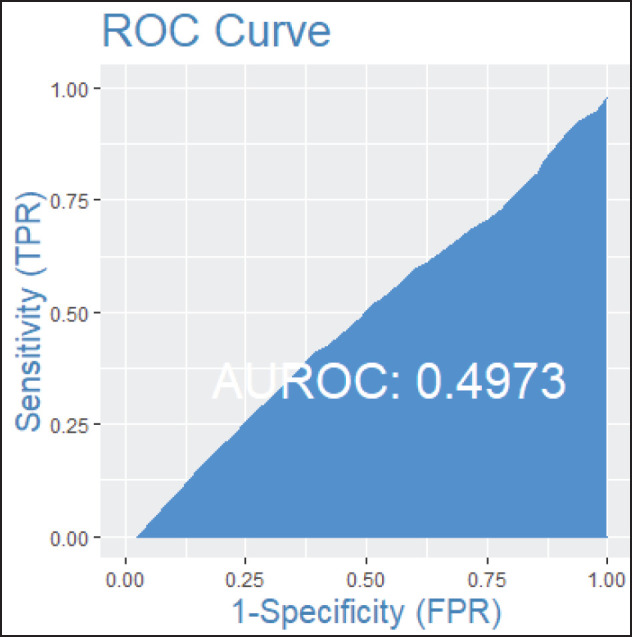
ROC curve in patients without lymph node involvement.

**Figure 2.**
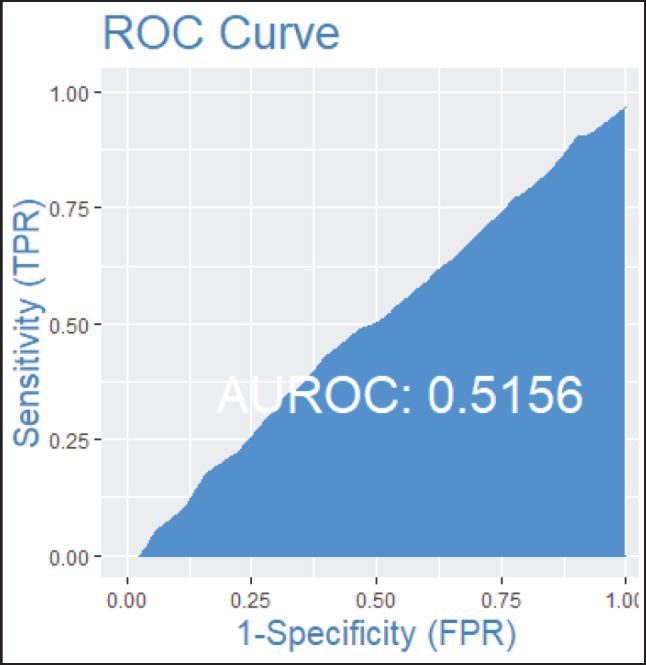
ROC curve by age groups: patients over 50 years old.

Among patients without lymph node involvement (*n* = 179), age, Ki67 values, and PRs predicted the RS after multivariate analysis. HER2-low status was not associated with differences in RS (*p* = 0.41). Using Ki67 cutoff points of 5%, 14%, 20% and 30%, respectively, 33%, 25%, 26% and 33% of patients were classified as Luminal A and had RS results associated with chemotherapy recommendation. Among postmenopausal patients with chemotherapy recommendations, 61% were classified as low risk according to the Tennessee Nomogram [10]. The predictive capacity of the Tennessee nomogram is evaluated in all included patients. Figures 1 and 2 (ROC curves) show the results based on nodal status and age. In none of the cases did the model demonstrate sufficient accuracy and efficacy in predicting genomic risk based on the clinical parameters used.

Among patients with lymph node involvement (*n* = 36), MonarchE inclusion criteria were not associated with a higher RS (*p* = 0.61). In Table 2, the results of the multivariate analysis are shown, considering both classic and new parameters.

Clinical risk (comprising tumour size and histological grade) showed marginal results in the tests and, as it had a *p*-value <0.1, was incorporated into the regression model. When patients were stratified into subgroups based on whether they were younger or older than 50 years, in the multivariate model, RP and Ki67 values were independent variables in predicting chemotherapy recommendation in those over 50 years. In those under 50 years, the only relevant variable for prediction was RP. HER2-low was not statistically significant (*p* = 0.19).

Patients with lymph node involvement were classified based on the potential indication of abemaciclib, and there were no significant differences regarding the potential indication for chemotherapy.

Secondary primaries were classified as tumours that developed either homolaterally or contralaterally to the primary tumour, and in the case of homolateral tumours, they had distinct histological characteristics from the previous one (lobular subtype versus ductal or previously being triple-negative or HER2+ tumours). Regarding findings related to secondary tumours based on chemotherapy recommendation, out of a total of 11 patients, the same criteria described by TailorX [7] and Sparano *et al* [2] for chemotherapy recommendation were applied.

**Figure 3.**
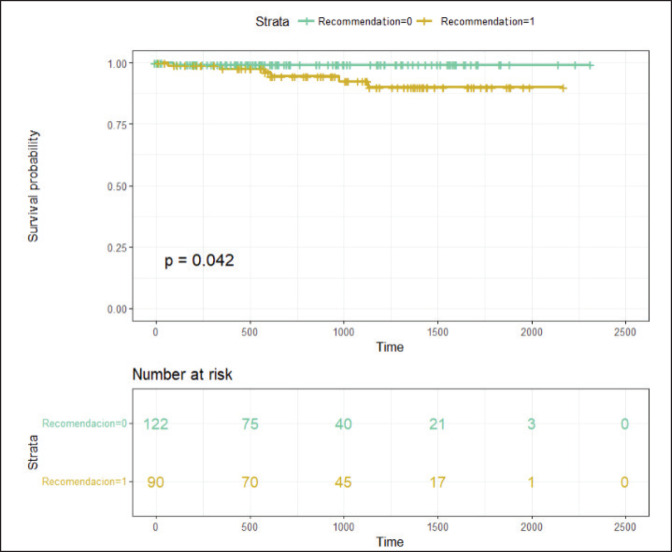
With a median follow-up of 26.93 months, Figure 3 displays the Kaplan-Meier curves of results in relapse-free survival according to chemotherapy indication. Recommendation 0 means NO indication for adjuvant chemotherapy, and Recommendation 1 means WITH recommendation for adjuvant chemotherapy.

There was a trend towards a higher RS in this subgroup of patients, although it did not reach statistical significance due to the small sample size. It was also observed that four of these patients had detected germline BRCA mutations, and three of them had high-risk tumours. With a median follow-up of 26.93 months (95% CI 21.6–33.06 months), the event-free survival in patients without chemotherapy recommendation was 99.1% (95% CI 97.5%–100%), whereas the event-free survival in patients with chemotherapy recommendation was 94.6% (95% CI 89.5%–99.9%) (Figure 3).

## Discussion

Hormone receptors (HRs) are prognostic and predictive factors for invasive breast cancer outcomes [16]. Regarding the accuracy of the HR percentage in correlating with prognosis, there is evidence from cohorts where it was observed that in luminal tumours, the absence of PR expression was associated with worse survival, with survival rates comparable to triple-negative tumours [17, 18]. This situation is infrequent and is observed in 2%–8% of cases. However, when analysing survival in estrogen receptor-negative (ER-) tumours with PR+, it is similar to ER+ tumours and better than ER- tumours with PR- [19].

Therefore, our work aligns with the literature reporting that in HR+ HER2- breast cancers, PR is an independent prognostic marker. PR expression in luminal breast cancer appears to depend on age and menopausal status, with lower expression rates in postmenopausal and older women [20]. According to the St. Gallen guidelines [1], both PR and Ki-67 are indicative of prognosis in HR+ HER2- tumours.

In relation to the positivity of HR and its linear correlation with recurrence risk, data from Marazzi *et al* [12] work evaluated 407 patients (54.55% over 50 years old), and logistic regression analysis showed that the RS score was significantly associated with ER (*p* = 0.004), PR (*p* < 0.0001), and Ki-67 (*p* < 0.0001). Generalised linear regression resulted in a model with an area under the curve of 0.92 (sensitivity 84.2%, specificity 80.1%).

As for other clinicopathological parameters, Crager *et al* [21] studied the correlation between Oncotype scores and the percentage of Ki-67 reported in 311 breast cancer tumour samples. They observed that among 218 samples with RS 0–25, 54 (25%) had Ki-67 ≥20%, and among 80 samples with RS 26–100, 23 (29%) had Ki-67 <20%, revealing a moderately positive correlation between the Oncotype DX breast cancer RS and Ki-67 by immunohistochemistry. This would imply that these pathological characteristics may have more prognostic weight in postmenopausal patients than in premenopausal ones. Our study was characterised by a smaller patient sample, and we noticed that some variables could predict RS adequately (such as PR). However, none of the other criteria had a statistically significant association regardless of age (over or under 50 years), making it less feasible to avoid genomic platforms in our scenario.

Regarding the negative results for concordance between adjuvant iCDK criteria and genomic risk, Sheffield *et al* [3] correlated each of the MonarchE clinical criteria (lymph node involvement, histological grade, tumour size >5 cm, and Ki-67 >20%) in a phase III study and evaluated invasive disease-free survival and distant recurrence-free survival in early HR+ HER2-negative breast cancer patients. They observed that the high clinical risk group (557 patients) had a higher risk of events than the low clinical risk group. The need to expand the adjuvant intensification indication in patients with genomic risk who did not meet the necessary clinical criteria is reflected in the treatment criteria of the phase III Natalee trial, which includes high genomic risk [22].

According to recent retrospective analysis publication results, HER2-low status could be prognostic for worse recurrence-free survival compared to HER2 negative patients [23]. However, in our cohort, HER2-low status was not prognostic for higher RS by platforms. On the other hand, Mutai *et al* [24] published that the prognostic value varies according to genomic risk results, where in high genomic risk patients, HER2-low status appears to be a positive prognostic factor compared to HER2 negative.

One of the limitations in low-resource countries like Argentina is the high cost of these genomic platforms. Cost-effectiveness evaluation studies exist; Vladislav *et al* [25] developed an analysis model comparing the Oncotype DX test with clinical risk tools in patients with 1 to 3 positive axillary lymph nodes, based on RxPONDER trial results. This analysis presented in terms of cost per quality-adjusted life years gained and found that the Oncotype DX test was more effective (with approximately 0.02 additional years of health-related life) at an estimated lower cost (−£989) compared to the use of clinical risk tools alone. It should be noted that this is a study of a small number of patients in a high-income region like the UK.

## Conclusion

In our real-world sample, the mere application of clinical and pathological parameters was insufficient to predict RS outcomes. Modern parameters, such as HER2-low status or adjuvant abemaciclib recommendations, were not associated with RS differences. A higher RS was observed in samples from secondary primary tumours. Further evidence is needed to understand if prior hormone therapy exposure impacts the biological risk of secondary primary tumours. Among the limitations of this study is the limited number of patients included in this retrospective study. This diminishes its statistical power to accurately judge a predictive model like a nomogram.

## Conflicts of interest

There are no conflicts of interest.

## Funding

None of the authors received funding for the development of this work or this manuscript.

## Figures and Tables

**Table 1.** Clinicopathological characteristics of the patients.

Characteristics	% (*N*)
Age	53 years
Positive lymph nodes	16.7% (36)
Negative lymph nodes	83.2% (179)
Under 50 years old	55.6% (119)
HER2-low	20% (43)
Luminal A	28.8% (62)
Luminal B	71.1% (153)
BRCA germline mutation	4.1% (9)
Patients meeting MonarchE inclusion criteria [4]	3.2% (7)

**Table 2.** Multivariate analysis considering both classic and new parameters.

Variable	*p*-value
Age	<0.001
Ki 67	<0.001
PR value	<0.001
Estrogen receptor value	0.045
Her 2 low status	0.169
Clinical risk (composed of size and histologic grade)	0.052
